# Delirium and dementia retrospective cohort study

**DOI:** 10.1192/j.eurpsy.2023.859

**Published:** 2023-07-19

**Authors:** O. Martin-Santiago, C. Alario-Ruiz, G. Guerra-Valera

**Affiliations:** Department of Psychiatry, Hospital Clinico Universitario de Valladolid, Valladolid, Spain

## Abstract

**Introduction:**

Delirium is common and is associated with many adverse short-term consequences as increased hospital costs, health care complications, and increased mortality. Long-term cognition consequences on delirium have not been well synthesized and quantified.

**Objectives:**

Our study aims to determine the relationship between an episode of delirium and subsequent dementia and death over five years.

**Methods:**

Postoperative delirium, previous psychiatric disorders, *mental health* service use, and *death data* collected from a cohort of inpatients diagnosed with delirium that requires psychiatric attendance in a general hospital were analyzed. Between 2009 and 2011, we started a follow-up of 91 patients aged 65 years or older at baseline for 60 months.

**Results:**

Five patients (5.4%) were diagnosed with dementia previously. During the first year, 35 patients without previous dementia (40.6%) died. More than half of the one-year survivors (27; 52.9%) were diagnosed with dementia at the follow-up. Differences in age (79,5 vs 80.3; Z=-0.07; p=0.93), survival time (54.8 vs 48.8;Z=1.30;p=0.19), postoperative delirium rates (74%vs66.6%; χ2=0.33, DF=1, p=0.56) and mental disorder antecedents were not found. Patients with dementia after delirium were more likely to be attentive in mental health services (48.1vs16.6%; χ2=5.666, DF=1, p=0.017).

**Conclusions:**

In our study, delirium is an important risk marker for dementia and death and is significantly associated with the long-term cognitive decline in surgical and non-surgical patients. Subsequent follow-up in *mental health* service could help detect dementia after episodes of delirium and lead to fewer potentially harmful interventions such as hospitalization or antipsychotic medication. An important question to determine is whether delirium is simply a risk marker for dementia or whether the delirium could cause neuronal damage leading to dementia.

**Disclosure of Interest:**

None Declared

